# Supplementary material for: Template-directed vertical photopolymerization for construction of triphenylamine-based poly(diacetylene) nanofibers
Source: Nat Commun. 2026 Feb 28;17:3731. doi: 10.1038/s41467-026-70114-x (PMC13103384; doi:10.1038/s41467-026-70114-x)
Supplement: Supplementary file 1 — Supplementary Infomation [file 41467_2026_70114_MOESM1_ESM.pdf]

Supplementary Information

Template-Directed Vertical Photopolymerization for Construction of Triphenylamine-Based Poly(diacetylene) Nanofibers

Yingbo Lu,[1,2,3] Luyao Jin,[1,2] Jiani Wang,[1,2] Qiang Fang,[4] Shuping Wang,[1,2] Jianying Huang,[4] Zibin Zhang,*[,1,2] Feihe Huang,[5,6,7] Shijun Li*[,1,2]

[1]Key Laboratory of Organosilicon Chemistry and Material Technology of Ministry of Education, College of Material, Chemistry and Chemical Engineering, Hangzhou Normal University, Hangzhou 311121, P. R. China.

[2]Zhejiang Key Laboratory of Organosilicon Material Technology, College of Material, Chemistry and Chemical Engineering, Hangzhou Normal University, Hangzhou 311121, P. R. China.

[3]College of Chemistry and Chemical Engineering, Central South University, Changsha 410083, P. R. China.

[4]College of Food Science and Biotechnology, Zhejiang Gongshang University, Hangzhou 310018, P. R. China.

[5]State Key Laboratory of Soil Pollution Control and Safety, Stoddart Institute of Molecular Science, Department of Chemistry, Zhejiang University, Hangzhou 310058, P. R. China.

[6]Key Laboratory of High-Performance Adhesion Functional Materials and Application Technology of Zhejiang, ZJU-Hangzhou Global Scientific and Technological Innovation Center, Zhejiang University, Hangzhou 311215, P. R. China.

[7]Zhejiang-Israel Joint Laboratory of Self-Assembling Functional Materials, ZJU-Hangzhou Global Scientific and Technological Innovation Center, Zhejiang University, Hangzhou 311215, P. R. China.

Correspondence and requests for materials should be addressed to Z.Z. (Email: zzhang@hznu.edu.cn) or S.L. (Email: l_shijun@hznu.edu.cn).

Contents

1. ADDITIONAL DATA S3
2. SYNTHESIS OF THE COMPOUNDS... S5
3. NMR SPECTRA OF THE COMPOUNDS S10

1. *Additional data*

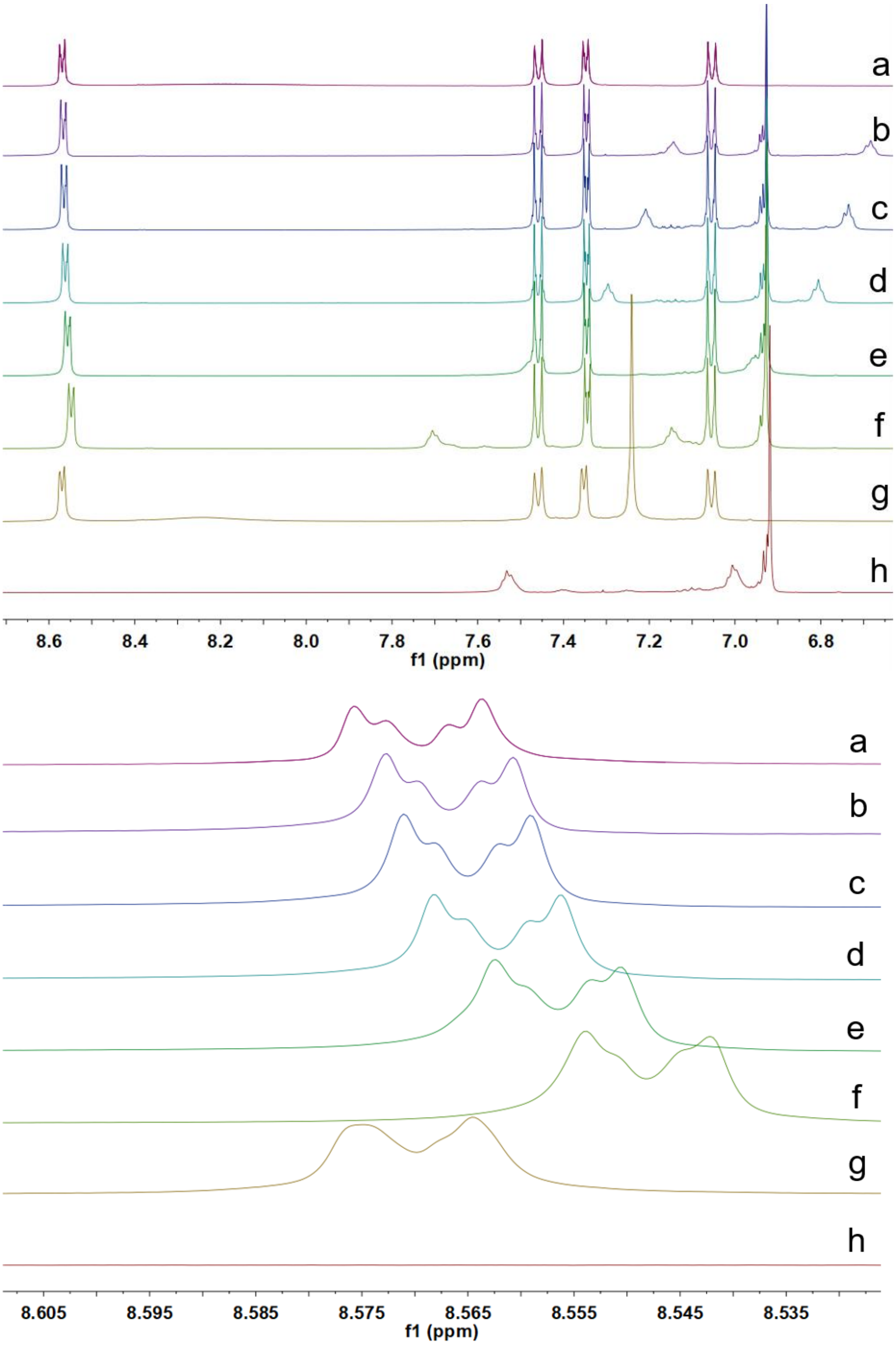

Supplementary Fig. 1 The partial ^{1}H NMR (500 MHz, CD_2Cl_2, 298 K) spectra of (a) **2** (20 mM); (b) **1** (3 mM) + **2** (1 mM); (c) **1** (7.5 mM) + **2** (2.5 mM); (d) **1** (15 mM) + **2** (5 mM); (e) **1** (30 mM) + **2** (10 mM); (f) **1** (60 mM) + **2** (20 mM); (g) **3** (60 mM) + **2** (20 mM); (h) **1** (60 mM).

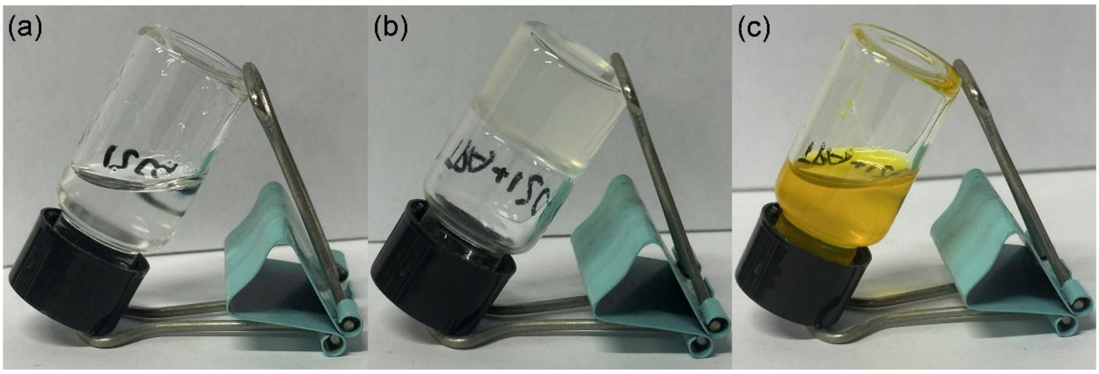

Supplementary Fig. 2 The Photographs of (a) **1** (1.5 mM) in MCH, (b) **1** (1.5 mM) + **2** (0.5 mM) in MCH and (c) **1** (1.5 mM) + **2** (0.5 mM) upon exposure to HCl for 1 min in MCH

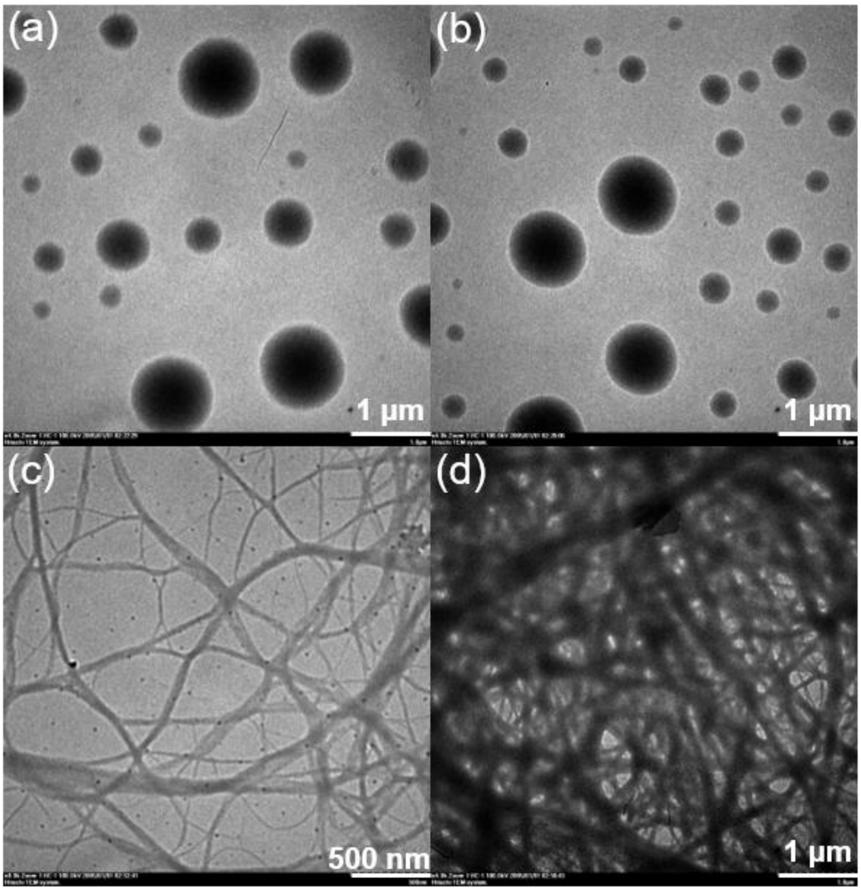

Supplementary Fig. 3 The TEM images of (a,b) **1** and (c,d) **1** + **2**.

2. *Synthesis of the compounds*

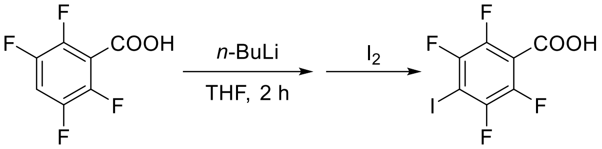

2,3,5,6-Tetrafluoro-4-iodobenzoic acid[1]: 2,3,5,6-Tetrafluorobenzoic acid (970 mg, 5 mmol) was dissolved in 80 mL dry THF. The solution was then cooled to –40 °C and *n*-butyllithium (2.5 M in hexane, 5 mL, 12.5 mmol) was added carefully. After stirred at –40 °C for 2 h, iodine (1650 mg, 6.5 mmol) was added and the reaction was further stirred at r.t. for 12 h. After completion of the reaction, HCl (2 M) was added and the organic layer was separated. The aqueous layer was extracted by diethyl ether, and the combined organic layer was washed with $Na_2S_2O_3$ solution, and brine. The solution was then dried over anhydrous $MgSO_4$, filtered, the solvent was evaporated. The product was obtained as a grey solid in 87% yield. ^{19}F NMR (471 MHz, $CDCl_3$, 298 K, ppm) δ 118.05, 135.91.

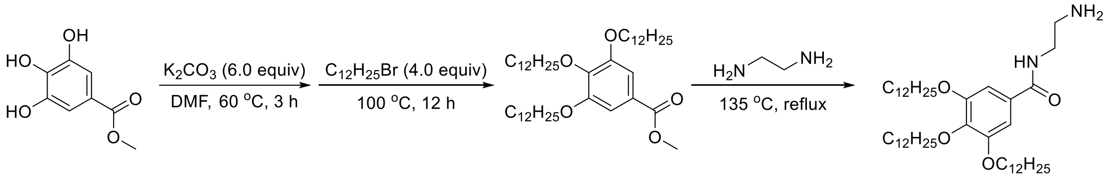

Methyl 3,4,5-tris(dodecyloxy)benzoate: Methyl 3,4,5-trihydroxybenzoate (5.1 g, 27.7 mmol) and potassium carbonate (23.8 g, 172.2 mmol) was placed in a two-necked flask, the reactant was vacuum purged and the flask was fulfilled with nitrogen. Followed by addition of DMF (150 mL), and the reaction was stirred for 2 hours at 60 °C. 1-Bromododecane (21.3 mL, 112.0 mmol) was then added slowly and the reaction was stirred at 80 °C for 12 hours. After completion of the reaction, the mixture was poured into icy water and the residue was collected by filtration and purified by silica gel chromatography (PE:EA = 200:1) to obtain **2** as a grey solid in 89% yield. ^{1}H NMR (500 MHz, $CDCl_3$) δ 7.25 (d, *J* = 5.9 Hz, 2H), 4.01 (td, *J* = 6.5, 3.8 Hz, 6H), 3.88 (s, 3H), 1.78 (ddd, *J* = 28.7, 14.4, 7.3 Hz, 6H), 1.52–1.42 (m, 6H), 1.39–1.21 (m, 50H), 0.88 (t, *J* = 6.9 Hz, 9H). ^{13}C NMR (126 MHz, $CDCl_3$) δ 166.9, 152.8, 142.3, 124.6, 108.0, 73.5, 69.1, 52.1, 31.9, 29.68, 29.65, 29.6, 29.38, 29.35, 26.1, 22.7, 14.1.

***N*-(2-aminoethyl)-3,4,5-tris(dodecyloxy)benzamide**: Methyl 3,4,5-tris(dodecyloxy)benzoate (15.0 g, 21.8 mmol) was dissolved in ethane-1,2-diamine (100 mL), and the reaction was refluxed at 135 °C for 48 hours. After completion of the reaction, the solvent was removed by using an evaporator,

and the crude product was purified by silica gel chromatography (DCM: MeOH: TEA = 200:2:1) to give the product as a grey solid in 48% yield. ^{1}H NMR (500 MHz, $CDCl_3$) *δ* 7.00 (s, 2H), 6.77 (s, 1H), 3.99 (dt, *J* = 13.0, 6.5 Hz, 6H), 3.51 (dd, *J* = 11.5, 5.7 Hz, 2H), 2.97 (t, *J* = 5.7 Hz, 2H), 1.83–1.70 (m, 7H), 1.45 (dd, *J* = 14.2, 8.5 Hz, 7H), 1.37–1.22 (m, 54H), 0.88 (t, *J* = 6.9 Hz, 9H).

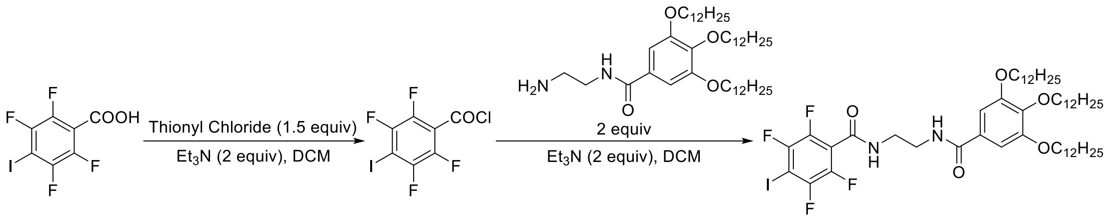

2,3,5,6-Tetrafluoro-4-iodo-*N*-(2-(3,4,5-tris(dodecyloxy)benzamido)ethyl)benzamide (1): 2,3,5,6-Tetrafluoro-4-iodobenzoic acid (1189.8 mg, 3.72 mmol) was dissolved in dry DCM (12 mL), thionyl chloride (0.33 mL) and Et_3N (1.0 mL) was added slowly to the solution and the suspension was stirred for 2 h. After evaporation of the solvent, the resulting residue was redissolved in 25 mL of dry DCM. *N*-(2-aminoethyl)-3,4,5-tris(dodecyloxy)benzamide (400.0 mg, 5.58 mmol) and Et_3N (1 mL) was added and the reaction was further stirred for 12 h. After completion of the reaction, icy water was added and the mixture was then extracted with DCM. The organic phase was collected and dried over anhydrous Na_2SO_4, and then filtered. The crude product was purified by silica gel chromatography (DCM: MeOH: TEA = 400:2:1) to give the product as a grey solid in 59% yield. ^{1}H NMR (500 MHz, $CDCl_3$) *δ* 8.09 (s, 1H), 7.48 (s, 1H), 6.93 (s, 2H), 3.95 (t, *J* = 6.5 Hz, 2H), 3.87 (t, *J* = 6.3 Hz, 4H), 3.57 (dd, *J* = 9.2, 4.4 Hz, 4H), 1.71 (dd, *J* = 16.7, 9.0 Hz, 6H), 1.48–1.37 (m, 6H), 1.27 (d, *J* = 18.2 Hz, 48H), 0.87 (t, *J* = 6.9 Hz, 9H). ^{13}C NMR (126 MHz, CDCl3) δ 168.5, 159.4, 152.9, 148.0 (d, *J* = 14.9 Hz), 146.0 (d, *J* = 15.4 Hz), 143.7 (d, *J* = 16.3 Hz), 141.6 (d, *J* = 16.3 Hz), 140.9, 128.3, 116.8 (t, *J* = 19.3 Hz), 105.2, 77.3, 77.0, 76.8, 73.4, 69.0, 53.3, 40.3, 40.1, 31.9, 30.3, 29.70, 29.68, 29.62, 29.55, 29.4, 29.33, 29.27, 26.0, 22.6, 14.0. HRMS (ESI) exact mass calculated for $C_{52}H_{83}F_4IN_2O_5$ ($[M + H]^+$): 1019.5356; found: 1019.5326.

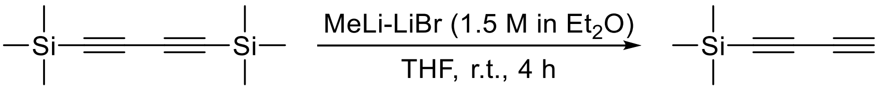

Buta-1,3-diyn-1-yltrimethylsilane[2]: 1,4-Bis(trimethylsilyl)buta-1,3-diyne (19.8 g, 102.0 mmol) was placed in a three-necked flask, the reactant was vacuum purged and the flask was fulfilled with nitrogen, then MeLi-LiBr (1.5 M in Et_2O, 71.4 mL, 107.1 mmol) and diethyl ether (200 mL) was added carefully. The solution was stirred for 4 h at r.t. and then cooled to 0 °C, followed with the

addition of methanol. The mixture was then extracted with saturated NH_4Cl solution and diethyl ether. The collected organic layer was washed with brine, dried over anhydrous Na_2SO_4, and filtered. Diethyl ether was removed by using a rotary evaporator with an ice bath. The product was obtained as a clear oil in 72% yield. ^{1}H NMR (500 MHz, $CDCl_3$) δ 2.11 (s, 1H), 0.21 (t, J = 3.5 Hz, 9H). ^{13}C NMR (126 MHz, $CDCl_3$) δ 87.4, 84.7, 68.3, 68.0, –0.6.

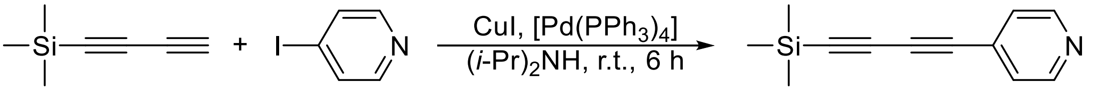

4-((Trimethylsilyl)buta-1,3-diyn-1-yl)pyridine [3]: Buta-1,3-diyn-1-yltrimethylsilane (7.0 g, 57.3 mmol), 4-iodopyridine (9.0 g, 44.0 mmol), copper(I) iodide (0.3 g, 1.5 mmol), tetrakis(triphenylphosphine)palladium (2.7 g, 2.4 mmol) was dissolved in 60 mL diisopropylamine. To remove dissolved oxygen, the reaction mixture was then performed three freeze–pump–thaw cycles, after which the flask was purged with nitrogen. After the reaction was stirred for 6 h at r.t., the mixture was filtered and the volatiles were removed by using a rotary evaporator. The crude product was purified by silica gel chromatography (CH_2Cl_2:Et_2O = 1:1) to give the product as a dark brown solid in 93% yield. ^{1}H NMR (500 MHz, $CDCl_3$) δ 8.34 (d, J = 6.1 Hz, 2H), 7.07 (d, J = 6.1 Hz, 2H), 0.00 (s, 9H). ^{13}C NMR (126 MHz, $CDCl_3$) δ 149.7, 129.6, 126.1, 93.5, 86.8, 78.3, 73.4, –0.7.

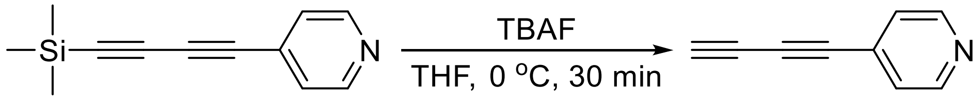

4-(Buta-1,3-diyn-1-yl)pyridine [4]: 4-((Trimethylsilyl)buta-1,3-diyn-1-yl)pyridine (0.8 g, 4.0 mmol) was dissolved in THF (160 mL), the solution was cooled to 0 °C and then tetrabutylammonium fluoride (1 M in THF, 4.4 mL) was added carefully. After stirred at 0 °C for 30 min, the reaction was treated with water. Subsequently, the resulting suspension was extracted with Et_2O. The organic phase was separated, dried over anhydrous Na_2SO_4, and then filtered. After the solvent was removed by a rotary evaporator, the crude product was purified by silica gel chromatography (CH_2Cl_2:Et_2O = 1:1) to give the product as a dark brown solid in 83% yield. ^{1}H NMR (500 MHz, DMSO-d_6) δ 8.60 (d, J = 6.1 Hz, 2H), 7.35 (d, J = 6.1 Hz, 2H), 2.59 (s, 1H).

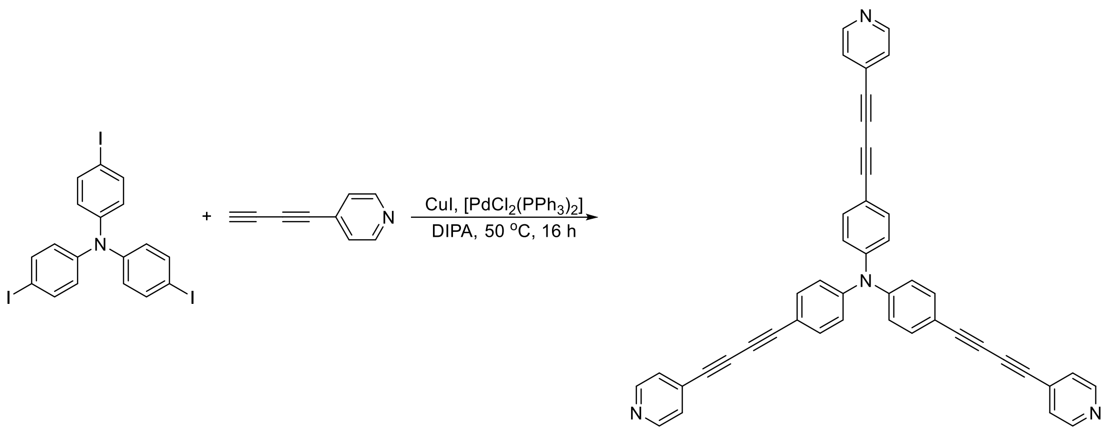

Tris(4-(pyridin-4-ylbuta-1,3-diyn-1-yl)phenyl)amine (2): Tris(4-iodophenyl)amine (269.5 mg, 0.43 mmol), 4-(buta-1,3-diyn-1-yl)pyridine (220.0 mg, 1.73 mmol), copper(I) iodide (27.2 mg, 0.14 mmol), trans-bis(triphenylphosphine)palladium(II) chloride (60.7 mg, 0.09 mmol) was dissolved in 65 mL of diisopropanolamine (DIPA). The mixture was stirred at 50 °C for 16 h. After completion of the reaction, the mixture was concentrated by a rotary evaporator, and the crude product was purified by silica gel chromatography (CH_2Cl_2:MeOH:TEA = 50:2:1) to give the product as a pale yellow solid in 83% yield. ^{1}H NMR (500 MHz, $CDCl_3$) δ 8.61 (dd, J = 4.6, 1.4 Hz, 6H), 7.46 (d, J = 8.7 Hz, 6H), 7.36 (dd, J = 4.5, 1.5 Hz, 6H), 7.06 (d, J = 8.7 Hz, 6H). ^{13}C NMR (126 MHz, $CDCl_3$) δ 149.9, 147.3, 134.1, 130.1, 126.0, 124.2, 116.2, 83.5, 78.6, 78.4, 73.4. HRMS (ESI) exact mass calculated for $C_{45}H_{24}N_4$ ([M + H]$^+$): 621.2074; found: 621.2008.

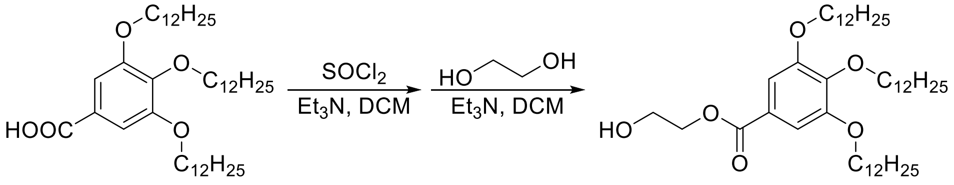

2-Hydroxyethyl 3,4,5-tris(dodecyloxy)benzoate: 3,4,5-Tris(dodecyloxy)benzoic acid (1450.0 mg, 2.15 mmol) was dissolved in dry DCM (7 mL), thionyl chloride (0.2 mL) and Et_3N (0.6 mL) was added slowly to the solution and the suspension was stirred for 2 h. After evaporation of the solvent, the resulting residue was redissolved in 15 mL of dry DCM. Ethane-1,2-diol (5.6 mL, 100.00 mmol) and Et_3N (0.6 mL) was added and the reaction was further stirred for 12 h. After completion of the reaction, icy water was added and the mixture was extracted with DCM. The organic phase was collected and dried over anhydrous Na_2SO_4, and then filtered. The crude product was purified by silica gel chromatography (PE: EA = 100:1) to give the product as a grey solid in 70% yield. ^{1}H NMR (500 MHz, $CDCl_3$) δ 4.49–4.41 (m, 2H), 4.01 (q, J = 6.6 Hz, 6H), 3.94 (d, J = 4.0 Hz, 2H), 1.85–1.70 (m, 6H),

1.50–1.44 (m, 6H), 1.37–1.23 (m, 50H), 0.88 (t, *J* = 6.9 Hz, 9H). ^{13}C NMR (126 MHz, $CDCl_3$) *δ* 166.9, 152.8, 142.7, 124.3, 108.2, 73.5, 69.2, 66.8, 61.6, 31.9, 29.71, 29.70, 29.68, 29.64, 29.62, 29.38, 29.35, 29.3, 26.1, 22.7, 14.1.

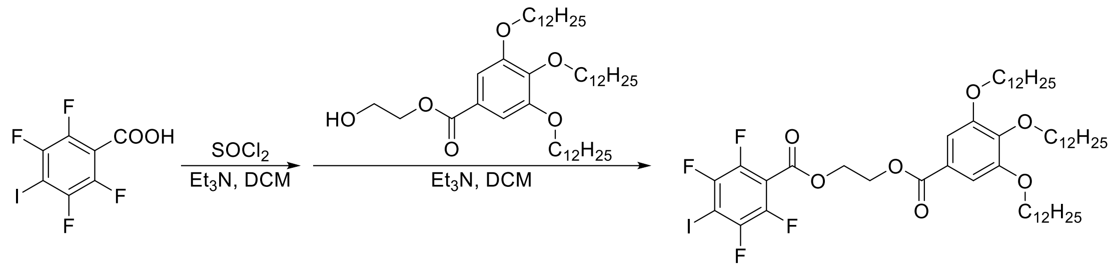

2-((3,4,5-tris(dodecyloxy)benzoyl)oxy)ethyl 2,3,5,6-tetrafluoro-4-iodobenzoate (3): 2,3,5,6-Tetrafluoro-4-iodobenzoic acid (360.00 mg, 1.13 mmol) was dissolved in dry DCM (3.5 mL). Thionyl chloride (0.11 mL) and Et_3N (0.33 mL) was added slowly to the solution and the suspension was stirred for 2 h. After evaporation of the solvent, the resulting residue was redissolved in 7 mL of dry DCM. 2-Hydroxyethyl 3,4,5-tris(dodecyloxy)benzoate (423.5 mg, 1.25 mmol) and Et_3N (0.33 mL) was added and the reaction was further stirred for 12 h. After completion of the reaction, icy water was added and the mixture was extracted with DCM. The organic phase was collected and dried over anhydrous Na_2SO_4, and then filtered. The crude product was purified by silica gel chromatography (PE: EA = 100:1) to give the product as a grey solid in 63% yield. ^1H NMR (500 MHz, $CDCl_3$) *δ* 7.25 (s, 2H), 4.74–4.66 (m, 2H), 4.63–4.55 (m, 2H), 4.00 (dt, *J* = 9.1, 6.5 Hz, 6H), 1.78 (dd, *J* = 24.2, 16.3 Hz, 6H), 1.45 (dd, *J* = 10.5, 4.6 Hz, 6H), 1.36–1.21 (m, 50H), 0.86 (t, *J* = 6.9 Hz, 9H). ^{13}C NMR (126 MHz, $CDCl_3$) δ 166.0, 159.2, 152.8, 148.4 (d, *J* = 14.6 Hz), 146.4 (d, *J* = 15.1 Hz), 145.1 (d, *J* = 16.9 Hz), 143.0 (d, *J* = 17.6 Hz), 142.6, 123.9, 112.8 (t, *J* = 15.9 Hz), 108.0, 73.5, 69.1, 64.3, 62.0, 31.9, 30.3, 29.9 – 29.5 (m), 29.5 – 29.1 (m), 26.04, 26.03, 22.7, 14.1. HRMS (ESI) exact mass calculated for $C_{52}H_{81}F_4IO_7$ ($[M + Na]^+$): 1043.4855; found: 1043.4787.

3. NMR spectra of the compounds

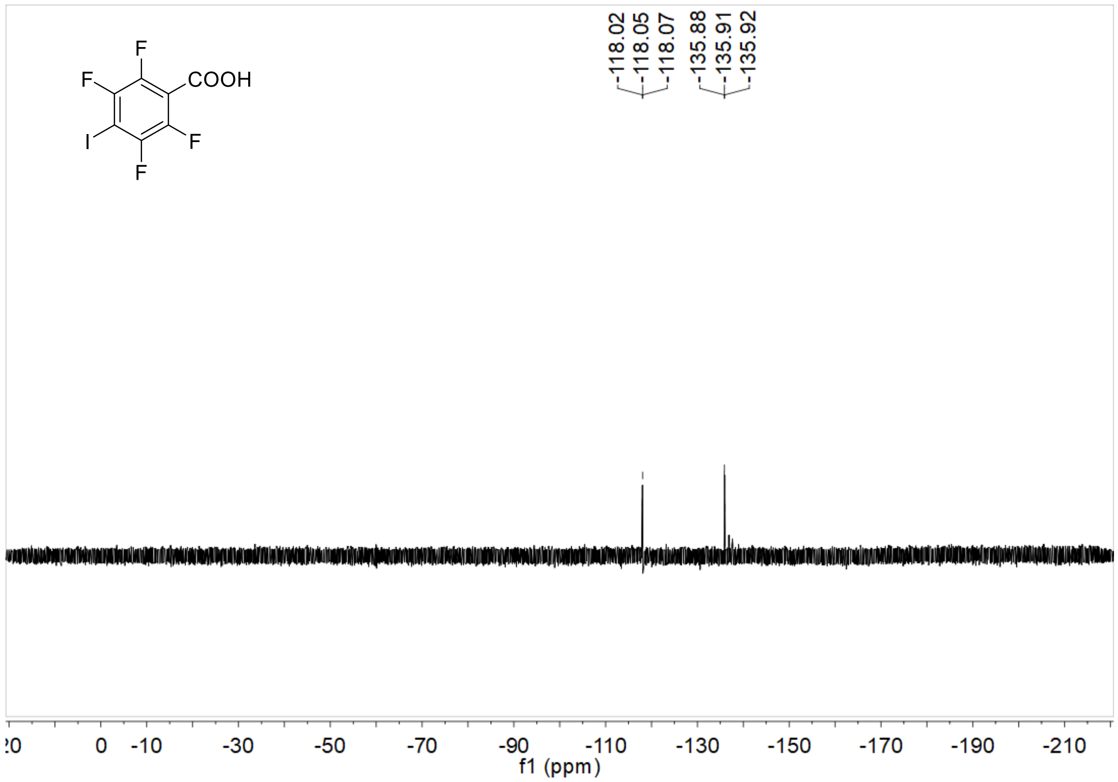

Supplementary Fig. 4 ^{19}F NMR (471 MHz, $CDCl_3$) spectrum of 2,3,5,6-tetrafluoro-4-iodobenzoic acid.

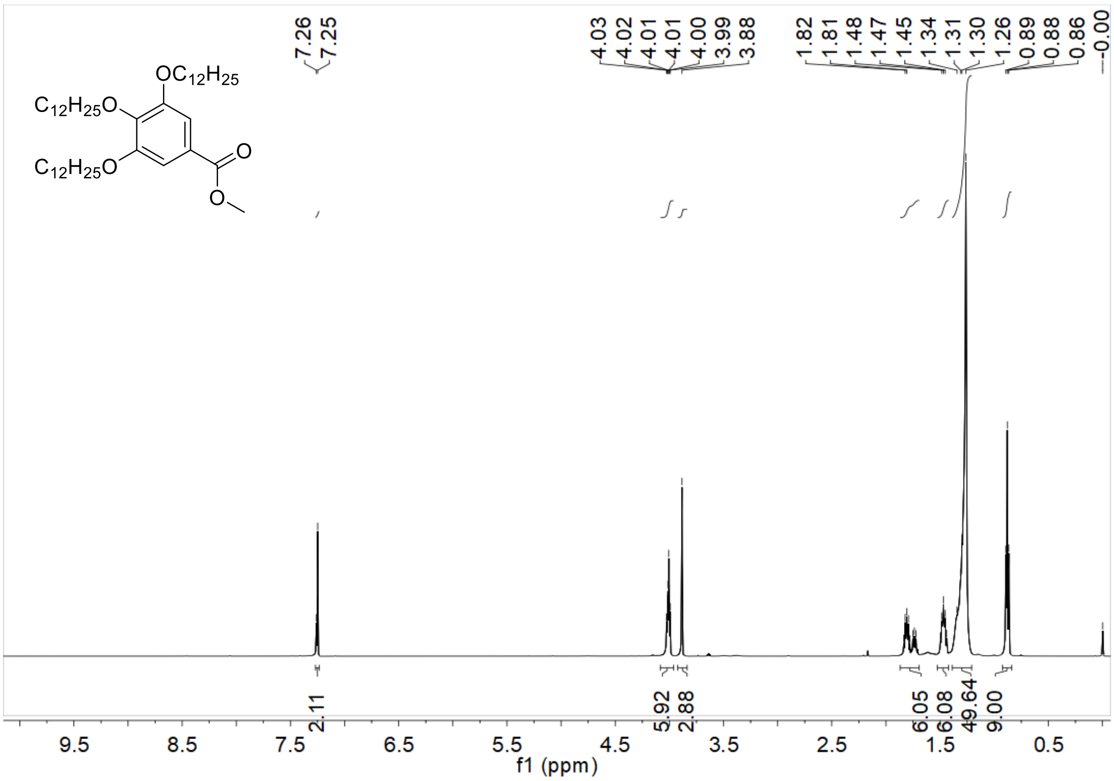

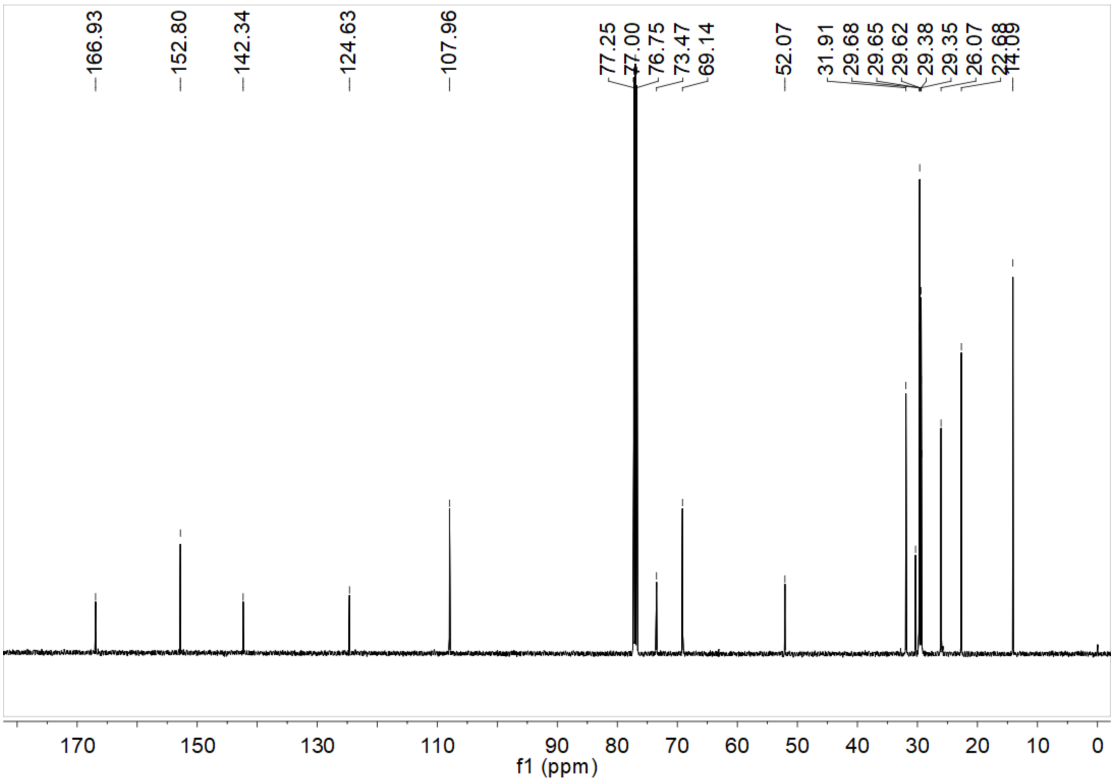

Supplementary Fig. 5 ^{1}H NMR (500 MHz, $CDCl_3$) and ^{13}C NMR (126 MHz, $CDCl_3$) spectra of Methyl 3,4,5-tris(dodecyloxy)benzoate.

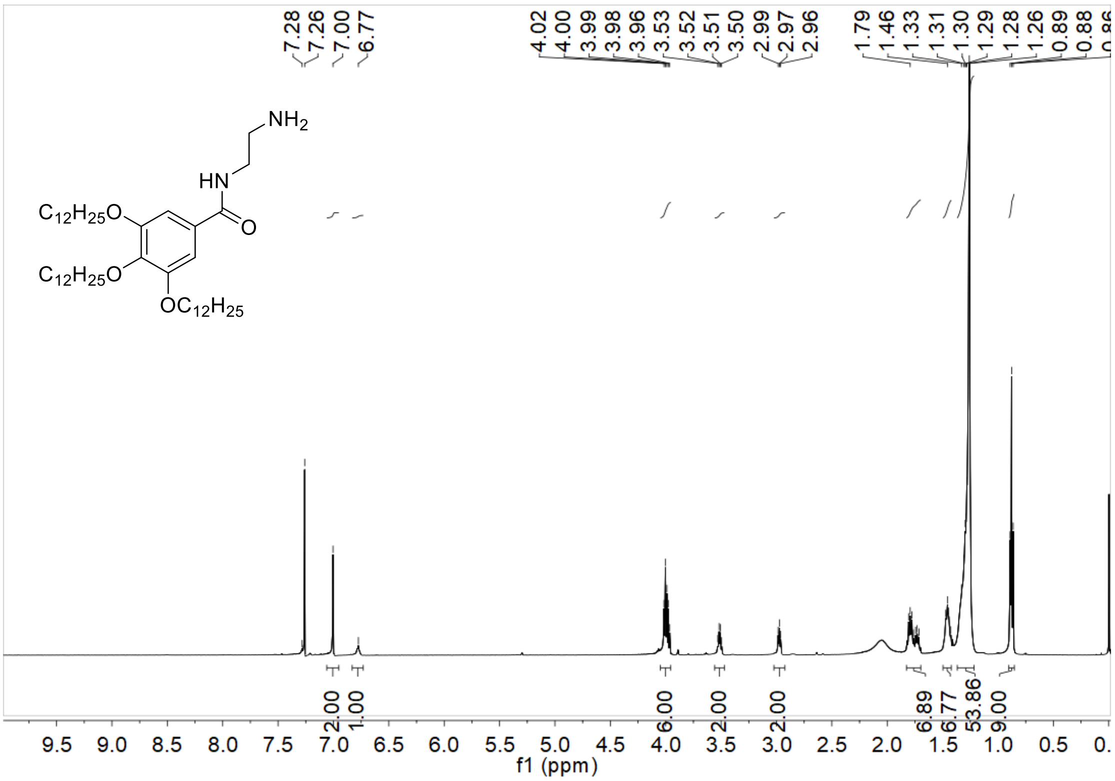

Supplementary Fig. 6 ^{1}H NMR (500 MHz, $CDCl_3$) spectrum of *N*-(2-aminoethyl)-3,4,5-tris(dodecyloxy)benzamide.

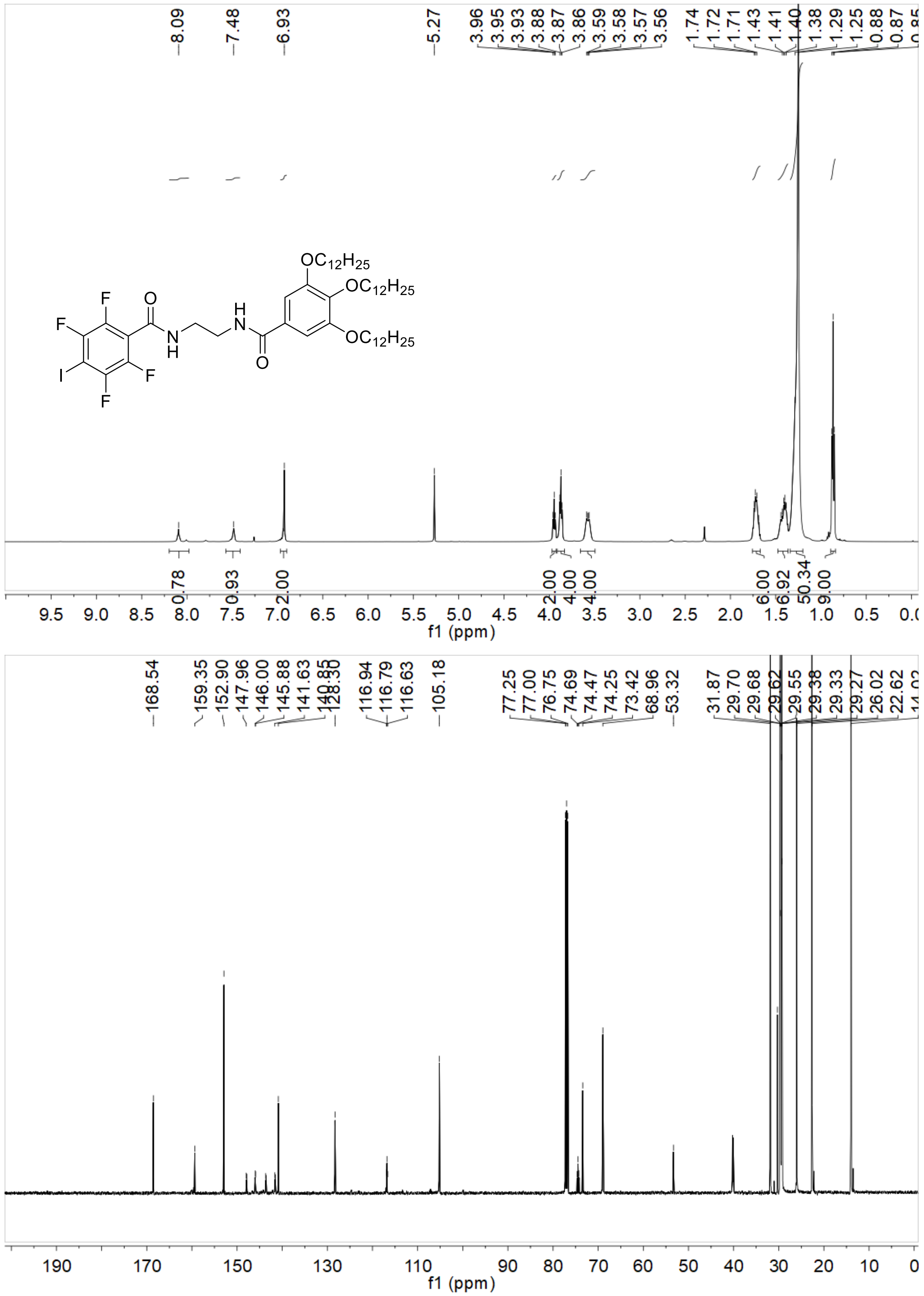

Supplementary Fig. 7 ^{1}H NMR (500 MHz, $CDCl_3$) and ^{13}C NMR (126 MHz, $CDCl_3$) spectra of 2,3,5,6-tetrafluoro-4-iodo-N-(2-(3,4,5-tris(dodecyloxy)benzamido)ethyl)benzamide (**1**).

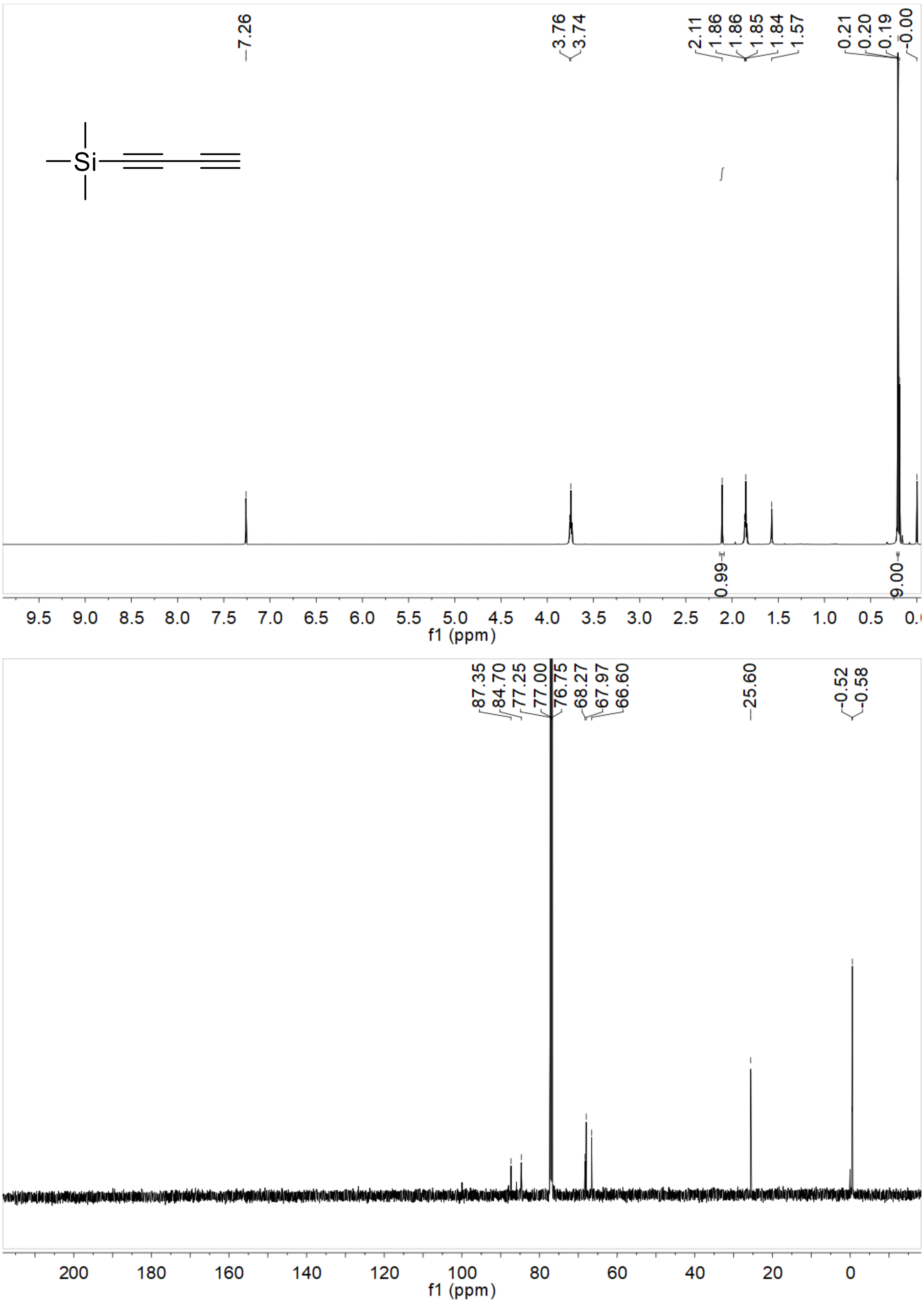

Supplementary Fig. 8 ^{1}H NMR (500 MHz, $CDCl_3$) and ^{13}C NMR (126 MHz, $CDCl_3$) spectra of buta-1,3-diyn-1-yltrimethylsilane.

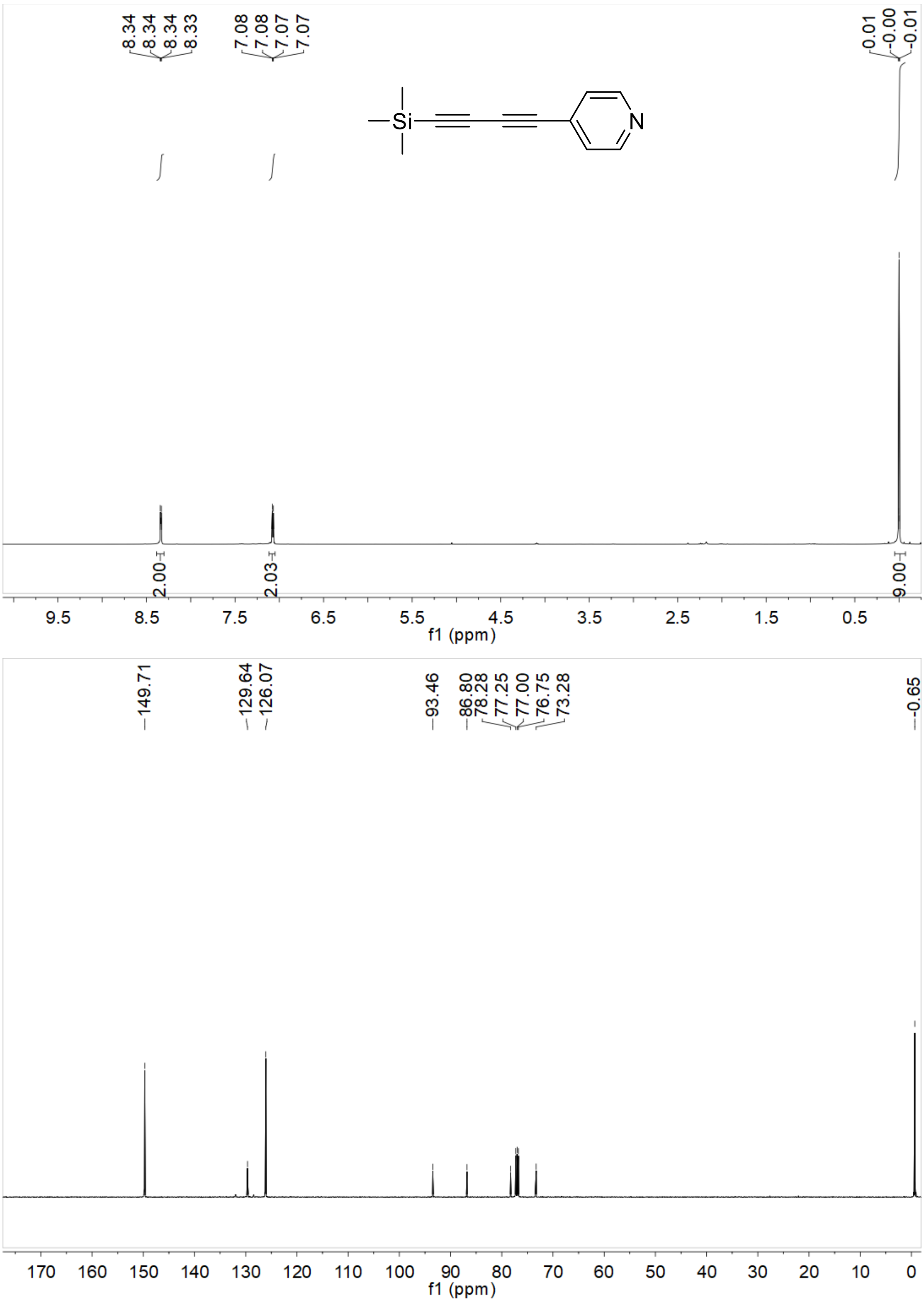

Supplementary Fig. 9 ^{1}H NMR (500 MHz, $CDCl_3$) and ^{13}C NMR (126 MHz, $CDCl_3$) spectra of 4-((Trimethylsilyl)buta-1,3-diyn-1-yl)pyridine.

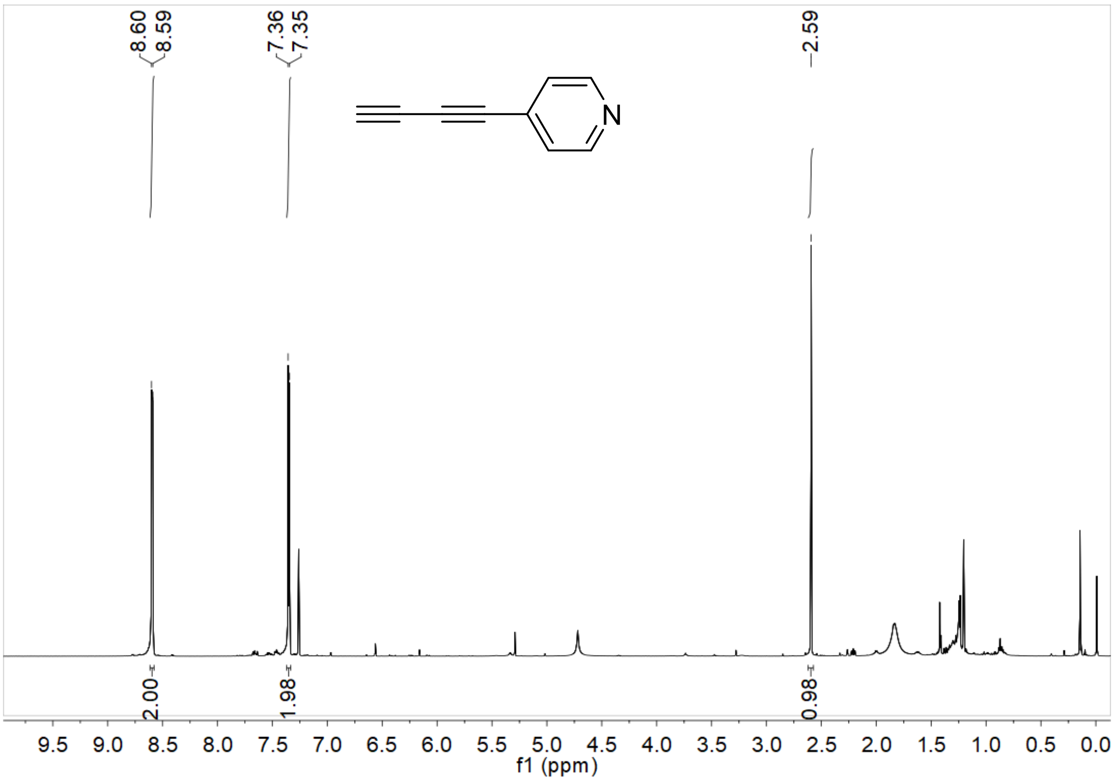

Supplementary Fig. 10 ^{1}H NMR (500 MHz, DMSO-d_6) spectrum of 4-(buta-1,3-diyn-1-yl)pyridine.

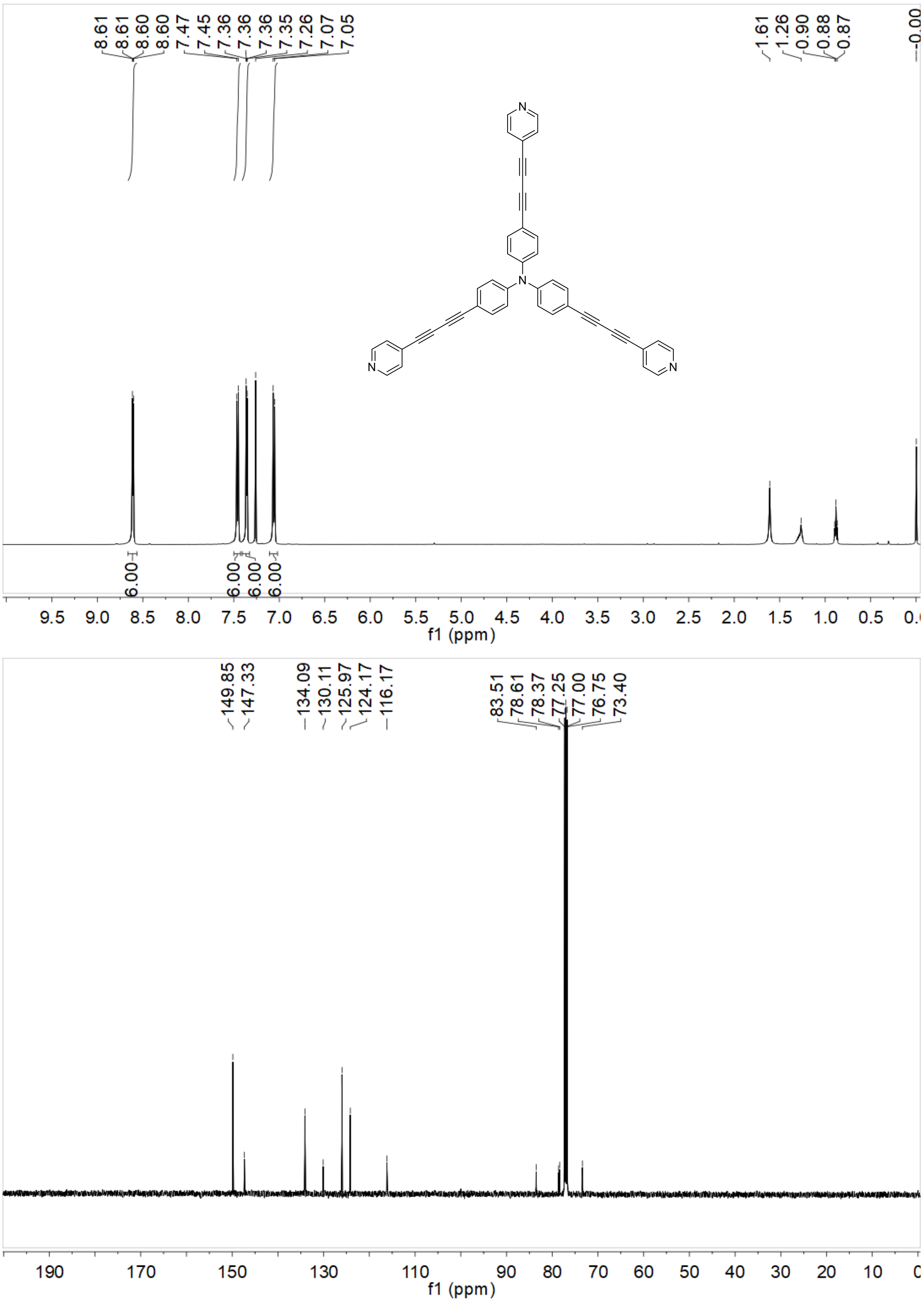

Supplementary Fig. 11 ^{1}H NMR (500 MHz, $CDCl_3$) and ^{13}C NMR (126 MHz, $CDCl_3$) spectra of tris(4-(pyridin-4-ylbuta-1,3-diyn-1-yl)phenyl)amine (2).

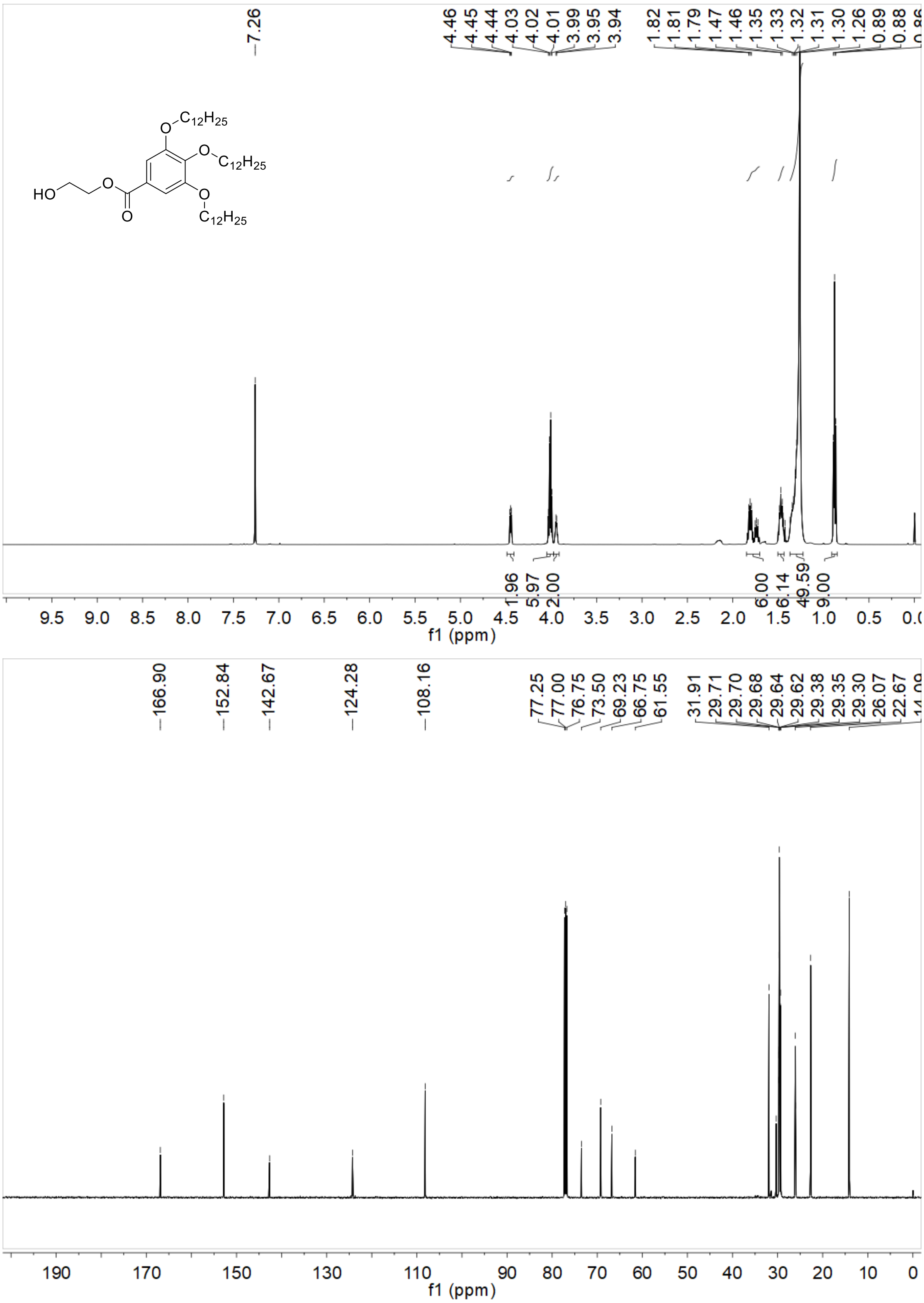

Supplementary Fig. 12 ^{1}H NMR (500 MHz, $CDCl_3$) and ^{13}C NMR (126 MHz, $CDCl_3$) spectra of 2-Hydroxyethyl 3,4,5-tris(dodecyloxy)benzoate.

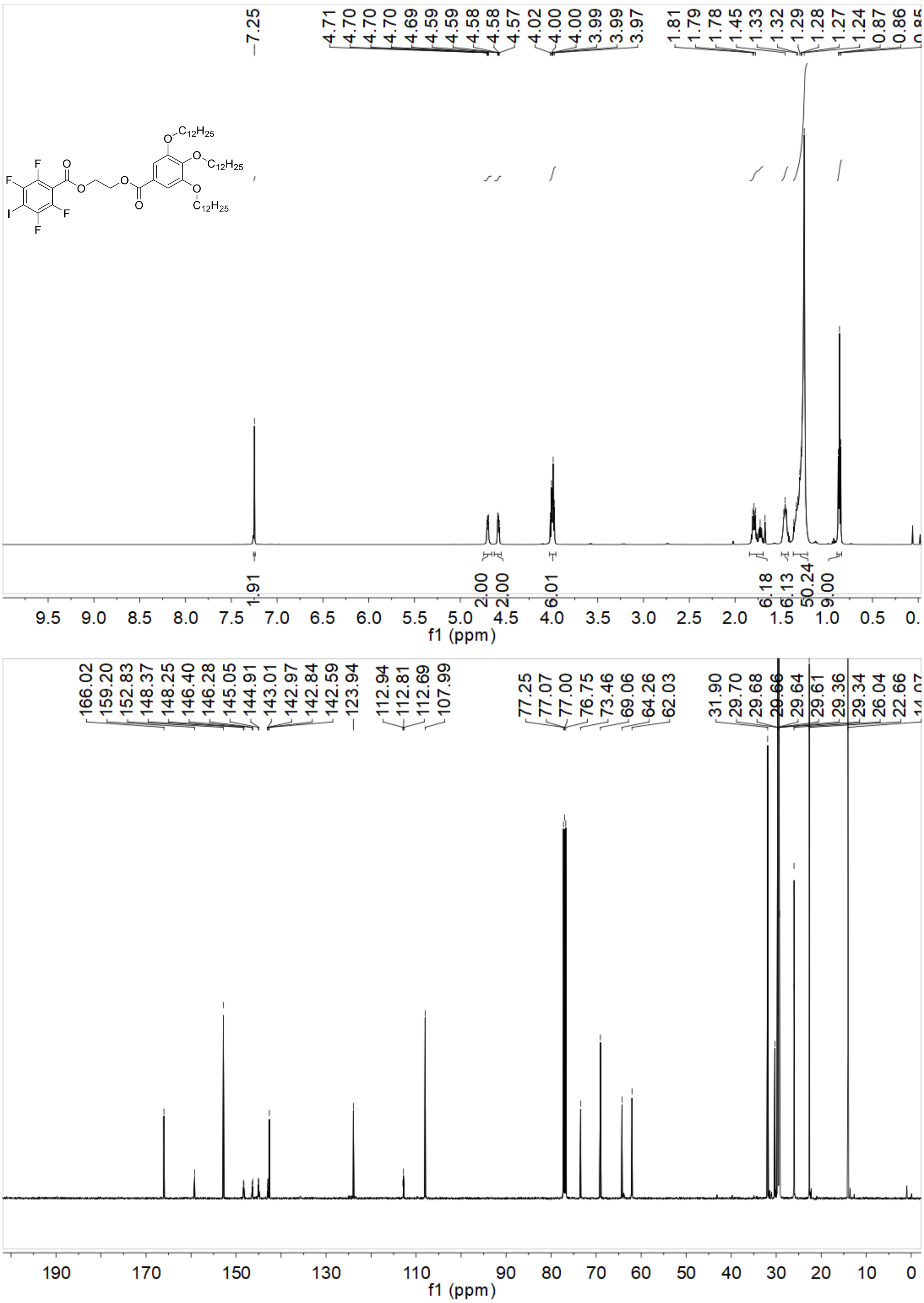

Supplementary Fig. 13 ^{1}H NMR (500 MHz, $CDCl_3$) and ^{13}C NMR (126 MHz, $CDCl_3$) spectra of 2-((3,4,5-tris(dodecyloxy)benzoyl)oxy)ethyl 2,3,5,6-tetrafluoro-4-iodobenzoate (3).

REFERENCES

[1] Hori, T., Kakinuma, S., Ohtsuka, N., Fujinami, T., Suzuki, T. & Momiyama, N. Synthesis of Halogen-Bond-Donor-Site-Introduced Functional Monomers through Wittig Reaction of Perfluorohalogenated Benzaldehydes: Toward Digitalization as Reliable Strategy in Small-Molecule Synthesis. *Synlett* **34**, 2455-2460 (2023).

[2] Farha, O. K., Wilmer, C. E., Eryazici, I., Hauser, B. G., Parilla, P. A., Oneill, K., Sarjeant, A. A., Nguyen, S. T., Snurr, R. Q. & Hupp, J. T. Designing Higher Surface Area Metal–Organic Frameworks: Are Triple Bonds Better Than Phenyls? *J. Am. Chem. Soc.* **134**, 9860-9863 (2012).

[3] Moreno-Garcia, P., Gulcur, M., Manrique, D. Z., Pope, T., Hong, W., Kaliginedi, V., Huang, C., Batsanov, A. S., Bryce, M. R., Lambert, C. & Wandlowski, T. Single-Molecule Conductance of Functionalized Oligoynes: Length Dependence and Junction Evolution. *J. Am. Chem. Soc.* **135**, 12228-12240 (2013).

[4] Iwasaki, M., Shichibu, Y. & Konishi, K. Unusual Attractive Au–π Interactions in Small Diacetylene-Modified Gold Clusters. *Angew. Chem. Int. Ed.* **58**, 2443-2447 (2019).
